# Commentary: Feasibility to estimate mean systemic filling pressure with inspiratory holds at the bedside

**DOI:** 10.3389/fphys.2023.1135769

**Published:** 2023-03-06

**Authors:** Per Werner Moller, David Christian Berger

**Affiliations:** ^1^ Department of Anaesthesia, SV Hospital Group, Institute of Clinical Sciences at the Sahlgrenska Academy, University of Gothenburg, Gothenburg, Sweden; ^2^ Department of Intensive Care Medicine, Inselspital, Bern University Hospital, University of Bern, Bern, Switzerland

**Keywords:** hemodynamics, venous return, mean systemic filling pressure, heart-lung interactions, guytonian model

In a recent study Wijnberge and colleagues assessed the ability of mean systemic filling pressure (MSFP) determined with the inspiratory-hold method (MSFP_insp_hold_) to track fluid boluses in 20 sedated and ventilated patients after coronary artery bypass grafting (Wijnberge et al., 2022). Since the method has been available for a decade, the authors implicitly raised the question why it has not been implemented in routine clinical use? The authors are to be commended to include a clinical feasibility assessment in their well performed study. Still, we think the main reason for the method not being widely used is not that it may be perceived as cumbersome, but rather that it has been proved inaccurate.

We share the opinion of the authors that bedside knowledge of stressed vascular volume and driving pressure for venous return (VRdP) could aid in managing haemodynamic therapy. We respect the impetus to find a practical solution, but experimental evidence suggests that it does not come in the form of MSFP_insp_hold_.

The development of the concept to assess MSFP by recording pressure-flow data pairs during changing inspiratory pressure is a fascinating story about applied physiology and heart-lung interactions and has deepened the understanding of venous return (VR) physiology. Already from the start, MSFP_insp_hold_ produced surprisingly high values of MSFP and VRdP [the record being 33 and 24 mmHg respectively (Persichini et al., 2012)] as compared to zero-flow measurements in animal models and patients [clinical testing of implantable cardioverter-defibrillator devices typically report MSFP and VRdP of 10–13 and 4–4.5 mmHg respectively (Jellinek et al., 2000; Schipke et al., 2003)]. The hope of being able to measure the underlying MSFP with clinically useful accuracy was severely questioned when we demonstrated that MSFP_insp_hold_ not only overestimated reference MSFP — but did so with a bias that varied with the volume state (Berger et al., 2016). Our initial porcine study was small but well-conducted, with intact circulation and reference method MSFP measured during intermittent right atrial balloon-occlusion. An additional physiological/mathematical analysis was published separately (Werner-Moller et al., 2019). The reason that MSFP_insp_hold_ overestimates zero-flow MSFP is likely the volume-state dependent caval vein flow-restoration that occurs during the hold manoeuvre, modulated by loading and unloading of upstream venous capacitance vessels, by pressure-flow dissociation from vascular collapse (in hypovolemia), and activation/opening of vascular waterfalls (in euvolemia, but not in hypo- or hypervolemia) — in addition to the fact that increasing intrathoracic pressure in itself increases zero-flow MSFP. In experimental animal models of veno-arterial ECMO, providing a perfectly equilibrated reference method MSFP, we could prove the back-pressure role of RAP and quantify volume shifts associated with dynamic changes in VR and CO caused by airway-pressure manoeuvres (Moller et al., 2017; Moller et al., 2018). These studies confirmed euvolemic MSFP and VRdP to be between 7 and 10 and around 5 mmHg, respectively, and that positive pressure inspiration of a normal tidal volume increased zero-flow MSFP by 0.4 mmHg. And good news for those like Wijnberge interested in implementing VR physiology into clinical work is that we have come quite far in validating Parkin´s mean systemic pressure analogue (P_msa_) (Moller and Parkin, 2022; Werner-Moller et al., 2022). This simple mathematical model allows the calculation of an estimate of MSFP without the need of an intervention and is therefore free from the clinical obstacles illustrated in Wijnberge’s study, such as the need for controlled ventilation and sedation, time consuming manual execution of manoeuvres, and possible clinical hazards in haemodynamically unstable situations.

We have provided a critical analysis of the strengths and shortcomings of individual studies that intended to pave the way for the clinical use of the inspiratory-hold method [available in detail in reference (Werner Möller, 2019), sections 1.8–1.8.5]. It became clear that although individual studies were of high quality, incorrect early conclusions were carried on to following investigators. In his landmark paper, Pinsky stated that *“Volume loading causes a parallel shift of the instantaneous venous return curve to the right without significantly changing its slope”* (Pinsky, 1984). This important conclusion was not supported by any quantitative data, and the method section lacked statistics that could test the possible agreement between ‘instantaneous P_ms_’ and zero-flow MSFP. In contrast, we found that volume state did influence the accuracy of instantaneous P_ms_ compared to MSFP determined at right atrial occlusion (Werner-Moller et al., 2019). Although Versprilles and Jansen quantified the ebb-flow tide effect of intrathoracic pressure on pulmonary blood volume, only their first study included data from changing global blood volumes - and then only during tidal breathing, as opposed to during inspiratory hold (Versprille et al., 1990; Versprille and Jansen, 1993). The authors reported on VR flow recovery during inspiratory pauses but did not study the effect of changing blood volume on this phenomenon. Of note, they observed that the inspiratory shift of pulmonary blood volume into the systemic circulation was significantly lower in hypervolemia than in normo- and hypovolemia.

Still to date, to the best of our knowledge, there is only one study testing the agreement between MSFP_insp_hold_ and a zero-flow reference method over changing volume states (Berger et al., 2016). As those results confirmed the known problem of overestimation and provided possible mechanistic explanations, we concluded the inspiratory-hold method to be unsuitable for research or clinical use. However, one should not grieve that MSFP_insp_hold_ ended up in the “did not work” section of medical history — it rests there in great company and the road was rich in insights.

If there is no evidence that we have missed, anyone still *advocating* the use of MSFP_insp_hold_ would be obliged to validate the method properly: 1) experimentally compare MSFP_insp_hold_ against a zero-flow reference method over changing volume state; 2) define and determine experimentally the clinically acceptable within-method precision of MSFP_insp_hold_; and most importantly 3) present a balanced discussion of the strengths and weakness of the method, using *all available relevant evidence*. A reasonable alternative could be to direct your efforts to the exploration of Parkin´s P_msa_. Most likely, you have a highly interesting data set from your previous study!
